# Single-Tubed Wild-Type Blocking Quantitative PCR Detection Assay for the Sensitive Detection of Codon 12 and 13 *KRAS* Mutations

**DOI:** 10.1371/journal.pone.0145698

**Published:** 2015-12-23

**Authors:** Jun-Fu Huang, Dong-Zhu Zeng, Guang-Jie Duan, Yan Shi, Guo-Hong Deng, Han Xia, Han-Qing Xu, Na Zhao, Wei-Ling Fu, Qing Huang

**Affiliations:** 1 Department of Laboratory Medicine, Southwest Hospital, Third Military Medical University, Chongqing, 400038, P. R. China; 2 Department of General Surgery, Southwest Hospital, Third Military Medical University, Chongqing, 400038, P. R. China; 3 Institute of Pathology and Southwest Cancer Center, Southwest Hospital, Third Military Medical University, Chongqing, 400038, P. R. China; 4 Department of Infectious Diseases, Southwest Hospital, Third Military Medical University, Chongqing, 400038, P. R. China; University of Navarra, SPAIN

## Abstract

The high degree of intra-tumor heterogeneity has meant that it is important to develop sensitive and selective assays to detect low-abundance *KRAS* mutations in metastatic colorectal carcinoma (mCRC) patients. As a major potential source of tumor DNA in the aforementioned genotyping assays, it was necessary to conduct an analysis on both the quality and quantity of DNA extracted from formalin-fixed paraffin-embedded (FFPE). Therefore, four commercial FFPE DNA extraction kits were initially compared with respect to their ability to facilitate extraction of amplifiable DNA. The results showed that TrimGen kits showed the greatest performance in relation to the quality and quantity of extracted FFPE DNA solutions. Using DNA extracted by TrimGen kits as a template for tumor genotyping, a real-time wild-type blocking PCR (WTB-PCR) assay was subsequently developed to detect the aforementioned *KRAS* mutations in mCRC patients. The results showed that WTB-PCR facilitated the detection of mutated alleles at a ratio of 1:10,000 (i.e. 0.01%) wild-type alleles. When the assay was subsequently used to test 49 mCRC patients, the results showed that the mutation detection levels of the WTB-PCR assay (61.8%; 30/49) were significantly higher than that of traditional PCR (38.8%; 19/49). Following the use of the real-time WTB-PCR assay, the Δ*C*_q_ method was used to quantitatively analyze the mutation levels associated with *KRAS* in each FFPE sample. The results showed that the mutant levels ranged from 53.74 to 0.12% in the patients analyzed. In conclusion, the current real-time WTB-PCR is a rapid, simple, and low-cost method that permits the detection of trace amounts of the mutated *KRAS* gene.

## Introduction

Human colorectal carcinoma (CRC) is one of the most common malignancies in worldwide countries including China. The World Health Organization estimates that 608,000 people die each year from clinical complications and metastasis associated with CRC [1]. Cetuximab and panitumumab are two approved monoclonal antibody-based therapeutic medicines that target the epidermal growth factor receptor (EGFR). These therapeutic agents have been used for the palliative treatment of human metastatic CRC (mCRC) since 2004 and 2007, respectively. Both antibodies are competitive antagonists of EGFR ligands and therefore impede ligand binding, receptor dimerization, and activation of the downstream MAPK, PI3K/AKT, and JAK/STAT pathways [2–4]. However, cetuximab and panitumumab only demonstrate response and disease stabilization rates of approximately 10% and 30%, respectively [5,6]. Serial clinical studies have indicated that the *KRAS* genotype should be considered when selecting mCRC patients as candidates for anti-EGFR therapy, with *KRAS* wild-type patients presenting with better clinical effects following associated treatments [7,8]. Because the analysis of *KRAS* codon 12 and 13 mutations is now standard practice prior to commencement of anti-EGFR therapy, the development of a reliable, fast and economical clinical assay to detect these mutations has become increasingly important. However, due to the heterogeneous nature of intra-tumor development, the mutated cancer cells are always in the minority in clinically available tissue samples because of the excess availability of wild-type DNA. Indeed, a recent study indicated that a higher-sensitivity KRAS mutation analysis method could help to identify patients who had poor responses to anti-EGFR antibody therapy in mCRC [9–12]. Therefore, the development of reliable and sensitive methods to detect low-abundance mutations associated with *KRAS* would be extremely useful determinants prior to the clinical application of anti-EGFR antibody therapies in mCRC.

In order to use tumor-specific somatic mutations as biomarkers for clinical oncology, the mutation must be detected in the presence of a large excess of non-mutated DNA from normal cells [13]. High sensitivity in relation to *KRAS* mutation assays is crucial in minimizing the risk of false negative results in tumor specimens containing low quantities of mutated DNA [14–16]. This has previously been reported to be of crucial importance in mCRC in relation to response prediction to anti-EGFR treatment [12] Until now, various methods have been applied to detect *KRAS* mutations [1,14–25]. These methods include PCR restriction fragment length polymorphism mapping (PCR-RFLP), conventional allele-specific PCR (AS-PCR), amplification refractory mutation system (ARMS), high resolution melting analysis (HRMA), dual priming oligonucleotides (DPO), allele-specific hydrolysis or dual hybridization probes, smart amplification process version 2 (SMAP 2), TaqMan allelic discrimination assay, pyrosequencing, next generation sequencing (NGS), BEAMing, IntPlex, and droplet digital PCR (dPCR). Apart from the latter three methods, most of the other methods display limited sensitivity, ranging from 1% to 5%, in relation to the detection of mutated *KRAS* alleles in the presence of a large excess of wild-type *KRAS* alleles. However, although the latter three methods displayed greater sensitivity (up to 0.0005%) in relation to the detection of rarely mutated *KRAS* alleles, some disadvantages limited the application of these methods in clinical oncology. The BEAMing technique requires pre-amplification of tumor DNA followed by a requirement for the emulsion to be broken down and beads to be processed allowing fluorescent tagging of the different alleles prior to analysis using flow cytometry [24,26]. The InPlex method requires allele-specific primers to conduct specific types of mutant analysis, and therefore only one mutation type from the 12 possible mutations associated with KRAS at codons 12 and 13 could be detected in a single tube [25,27,28]. The droplet dPCR is still relatively expensive (i.e. up to $30 per sample) and time-consuming, and requires a specialized emulsion droplet dPCR instrument (e.g. QX100 or QX200 ddPCR system of Bio-Rad Laboratories, RainDrop Digital PCR of RainDance company) which is not commonly available in most clinical laboratories [13,22,29,30]. In previous publications, various assays using clamp-PCR strategies have been developed to detect *KRAS* mutations [31–34]. In the *KRAS* clamp-PCR assay, oligonucleotides including peptide nucleic acid (PNA), locked nucleic acid (LNA), and LNA/DNA chimeras were used to selectively inhibit the amplification of wild-type *KRAS*, thereby selectively amplifying mutant *KRAS* alleles and increasing associated sensitivities (up to 0.01%) [31–34]. In our laboratory, a wild-type-blocker PCR (WTB-PCR) assay was developed to ultrasensitively analyze rare *KRAS* gene mutations in formalin-fixed paraffin-embedded (FFPE) sections from mCRC patients [33]. However, agarose gel electrophoresis and Sanger sequencing were still required to reveal the mutation status of *KRAS* codons 12 and 13 [33]. In the present study, real-time WTB-PCR required primers that overlapped with the wild-type blocker (WTB) sequence in detection analysis. Hydrolysis fluorescent probes were developed to quantitatively analyze all 12 possible missense mutations associated with *KRAS* codons 12 and 13 in a single closed tube. The results indicated that the associated method permitted the ultrasensitive detection of *KRAS* mutations with up to 0.01% sensitivity. Therefore, WTB-PCR is likely to be a powerful tool that can be used in the response prediction of anti-EGFR therapy for mCRC patient treatment.

## Materials and Methods

### Patients and tissue samples

This study was approved by the institutional review board and ethics committee of Southwest Hospital (Chongqing, China). All patients were selected from an ethnic Chinese population, and written informed consent was obtained from each patient or their family members. To ensure that a sufficient quantity of tumor DNA could be extracted, assessment by hematoxylin-eosin (HE) staining was firstly performed to select FFPE tissue blocks that harbored at least 70% neoplastic cells. This was conducted to reduce the presence of non-neoplastic cells, thereby avoiding macrodissection tissue section [35]. Subsequently, 10 consecutive serially unstained sections (5 μm thick) were prepared from each FFPE tissue block, satisfying the aforementioned parameters. Both the first and tenth sections were further assessed by HE staining to avoid samples containing greater than a 10% difference in tumor cell content between the two aforementioned sections. The other eight sections were used for DNA isolation. Consecutive sections of each tissue block were mounted on eight glass slides (one section per slide) to facilitate the generation of 20 FFPE tissue samples from a total of 50 mCRC patients. Consecutive sections attained from each tissue block were stored in four sterile centrifuge tubes (two sections per tube) for another 30 samples.

### Genomic DNA extraction from FFPE sections

Genomic DNA (gDNA) from the FFPE sections was extracted using a QuickExtract^™^ FFPE DNA Extraction Kit (Cat. QEF81805; Epicentre Biotechnologies, Madison, WI, USA), a QIAamp^®^ DNA FFPE Tissue Kit (Cat. 56404; Qiagen, Valencia), a Paraffin Sample DNA Extraction Wax-Free^™^ DNA Kit (Cat. WF-100; TrimGen, Sparks, MD, USA), and a GTpure^™^ FFPE Tissue DNA Extraction Kit (Cat. 536–050; Gene Tech, Shanghai, China), respectively. The manufacturer’s instructions were followed during use of each of the kits. To generate comparative results, each aforementioned kit was used to extract FFPE DNA from two sections mounted on two glass slides or stored in a single tube for each sample. The gDNA of *Sus scrofa* (pig, porcine), human whole blood, and AsPC-1 cell lines was extracted with the QIAamp^®^ DNA Blood Mini Kit (Qiagen). DNA concentrations were accurately quantified using the Quant-iT PicoGreen dsDNA Assay Kit (Invitrogen, Eugene, OR, USA).

### Determination of remnant PCR amplification inhibitors in FFPE DNA solutions

The PCR mixture (20 μl) that was used to target porcine mitochondrial DNA sequences contained the following components: 1X Premix *Ex Taq* Master Mix (Perfect Real Time; TaKaRa, Dalian, China), 200 nM of forward primer (SW-171), 200 nM of reverse primer (SW-172), 50 nM of TaqMan probes (SW-301), 1 μl of each FFPE DNA solution, and 1 ng of porcine gDNA. The primer and probe sequences are listed in Table 1. In order to normalize the quantification cycles (*C*_q_) for each sample that had been spiked with porcine gDNA, reaction mixtures that contained no PCR amplification inhibitors and only 1 ng of porcine gDNA and 50 ng of human genomic DNA were used. Reactions were performed on a LightCycler 1.2 (Roche, Indianapolis, IN, USA) using the following cycling conditions: an initial denaturation step at 95°C for 20 sec followed by 40 cycles of 95°C for 5 sec, and 60°C for 30 sec.

**Table 1:** Sequences of Oligonucleotides used in the present study*.

Oligo ID	Oligo sequences (5' → 3')
SW-131	TTAAGCGTCGATGGAGGAGTT
SW-132	AGAATGGTCCTGCACCAGTAATA
SW-301	(FAM)ACTAGCCCCATTATCAGTACTATGCCA(BHQ1)
SW-145	AAGGTACTGGTGGAGTATTTGATAGTG
SW-428	GTATCGTCAAGGCACTCTTGCCTACGCCAC**g**AGCTC
SW-429	GTATCGTCAAGGCACTCTTGCCTACGCCAC**a**AGCTC
SW-430	GTATCGTCAAGGCACTCTTGCCTACGCCAC**t**AGCTC
SW-431	GTATCGTCAAGGCACTCTTGCCTACGCCA**g**CAGCTC
SW-432	GTATCGTCAAGGCACTCTTGCCTACGCCA**a**CAGCTC
SW-433	GTATCGTCAAGGCACTCTTGCCTACGCCA**t**CAGCTC
SW-434	GTATCGTCAAGGCACTCTTGCCTACGC**g**ACCAGCTC
SW-435	GTATCGTCAAGGCACTCTTGCCTACGC**a**ACCAGCTC
SW-436	GTATCGTCAAGGCACTCTTGCCTACGC**t**ACCAGCTC
SW-437	GTATCGTCAAGGCACTCTTGCCTACG**g**CACCAGCTC
SW-438	GTATCGTCAAGGCACTCTTGCCTACG**a**CACCAGCTC
SW-439	GTATCGTCAAGGCACTCTTGCCTACG**t**CACCAGCTC
SW-146	GATTTACCTCTATTGTTGGATCATATTC
SW-395	ATGTGTGACATGTTCTAATATAGTC
SW-397	CAAGGCACTCTTGCCTACG
SW-144	TACGCCACCAGCT(PO4)
SW-409	(FAM)CAACTACCACAAGTTTATATTCAGTCAT(BHQ-1)
SW-171	CCAAGGCATTTCACTACAAG
SW-172	GTTTGGGTTGATTGATTGTG

*In these reaction mixtures, the SW-145 universal forward primer and mutant allele specific reverse primers SW-428 to -439 were used to generate amplicons containing c.34G>C, c.34G>T, c.34G>A, c.35G>C, c.35G>T, c.35G>A, c.37G>C, c.37G>T, c.37G>A, c.38G>C, c.38G>T, and c.38G>A, respectively. The lower letters indicate the targeted alleles and the underlined sequences are those sequences that are identical to SW-397 and were used in reverse primers for the real-time PCR with or without WTB. The underlined letters in SW-144 indicate the LNA. The fluorescent reporter and quencher are indicated at the terminal sequences of SW409 and SW-301.

### Preparation of quality control (QC) plasmids

Nested PCR was used to prepare a QC plasmid to evaluate real-time PCR targeting of the *KRAS* gene. The PCR reaction mixture (20 μl) contained 1X *Ex Taq* Buffer (Mg^2+^ plus; TaKaRa), 250 μM of each dNTP (TaKaRa), 0.5 units of TaKaRa *Ex Taq* HS DNA Polymerase (TaKaRa), 200 nM each of SW-131 and SW-132, 50 ng of human homozygous wide-type gDNA (WT-gDNA). Reactions were performed on Veriti^™^ 96-well Thermal Cyclers (Applied Biosystems, Forster City, CA, USA) using the following cycling conditions: denaturation at 94°C for 5 min; 35 cycles at 94°C for 30sec, 60°C for 30 sec and 72°C for 30 sec; and a final extension step at 72°C for 5 min. The PCR products were diluted 50-fold and used in a second round of PCR. The second round of PCR contained the same reagents apart from the template and primers i.e. 200 nM each of universal forward (SW-145) and mutant-allele specific revers primers (SW-428 and SW439) (Table 1). The PCR products were sequenced using the BigDye Terminator V3.1 Cycler Sequencing Kit (Applied Biosystems). After the genotype of the amplicons was confirmed, the PCR products were cloned to construct the wild-type QC (WT-QC) and mutant QC (MT-QC) plasmids.

### Design of real-time WTB-PCR

**Fig 1:**
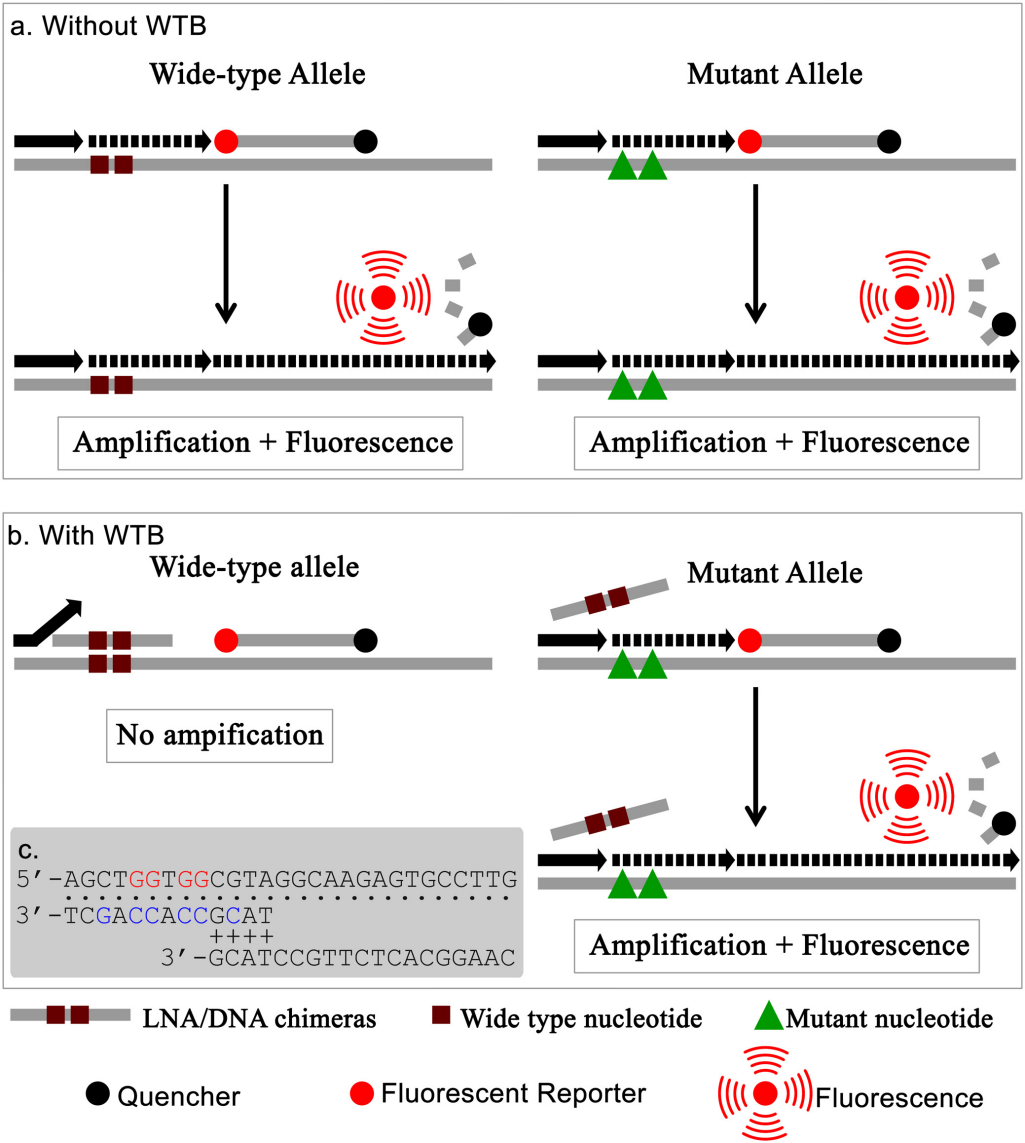
Principle of real-time WTB-qPCR. a. Real-time traditional PCR (i.e. without WTB) cannot distinguish WT- and MT-alleles of the *KRAS* gene as both forms release fluorescence upon amplification. b. Conversely, for real time WTB-PCR, only MT-alleles can be amplified because the amplification of WT-alleles is blocked by the WTB oligonucleotide. c. Demonstration of the overlapping sequences associated with WTB and reverse primer. The WT-allele template, WTB, and primer are shown at the top, middle, and bottom positions, respectively, in which the dot symbols indicate the complementary positions between the template and WTB or reverse primers, and the plus symbols indicate the overlapping sequences between WTB and reverse primers. The red and blue letters indicate WT-alleles in the template and LNA in WTB, respectively.

In order to efficiently and sensitively detect the rare codon mutations for codons 12 or 13 of *KRAS*, a LNA/DNA chimera oligonucleotide [33] (entitled “WTB” in the present study), was designed to specifically inhibit the amplification of *KRAS* sequences containing wild-type codons 12 and 13 (Fig 1). WTB was complementary to a region of the wild-type *KRAS* gene containing any one of the 12 possible missense mutations at codons 12 or 13. In both traditional PCR (Fig 1a) and WTB-PCR (Fig 1b), both forward and reverse primers targeted the sequences of the WT- and MT-alleles. However, the binding position of the 3'-end of the reverse primer overlapped with that of the WTB oligonucleotide. Using traditional PCR, both WT- and MT-alleles were amplified, resulting in corresponding fluorescent signals. As a result, the traditional PCR approach was not able to distinguish between WT- and MT-alleles. In the WTB-PCR, the WTB oligonucleotide was designed to be complementary to a region of wild-type *KRAS* sequence that can contain missense mutations at both codons 12 and 13. In order to avoid the extension of WTB by DNA polymerase in the PCR reactions, a phosphorylation group was modified at the 3'-terminus. The WTB oligonucleotide facilitated tight binding to *KRAS* containing both WT- and MT-alleles. However, a single base mismatch to MT-alleles (e.g. c.35G>A) would be expected to significantly decrease the blocking affinity, thereby allowing the amplification of *KRAS* MT-alleles.

Amplification of the KRAS gene with (WTB-PCR) or without WTB (traditional PCR) using TaqMan real-time PCR

Both real-time WTB-PCR and traditional PCR were performed in a final volume of 20 μl. The PCR reaction mixture contained 1X master mix containing Premix *Ex Taq* (Perfect Real Time; TaKaRa), 200 nM each of forward (SW-395) and reverse (SW-397) primers, and 50 nM of TaqMan probe (SW-409). These mixtures were subjected to amplification with (WTB-PCR) or without (traditional PCR) WTB (i.e., SW-144) using a series of final concentrations of SW-144 (i.e., 5.0, 2.0. 1.0 and 0.5 μM Table 1). Specific concentrations of human gDNA (i.e., 50 and 100 ng each 20 μl of reaction mixture) or a series of mutant-allele percentages (i.e., 100%, 50%, 25%, 10%, 1%, 0.1%, 0.01%) were also used in these reactions. In each of these experiments, the aforementioned QC plasmid was used as a positive control. Reactions were performed on a Mx3000P (Stratagene, La Jolla, CA, USA) with a 2 min pre-incubation step at 98°C followed by 40 to 55 amplification cycles of 5 sec at 98°C and 20 sec at 60°C. DNA sequencing was performed on both the traditional PCR and WTB-PCR products as previously described.

### Statistic analysis

One-way Analysis Of Variance (ANOVA) was used to analyze the properties of the commercially available FFPE DNA extraction kits.

## Results

### Comparative analysis of four commercially available FFPE DNA extraction kits

**Fig 2:**
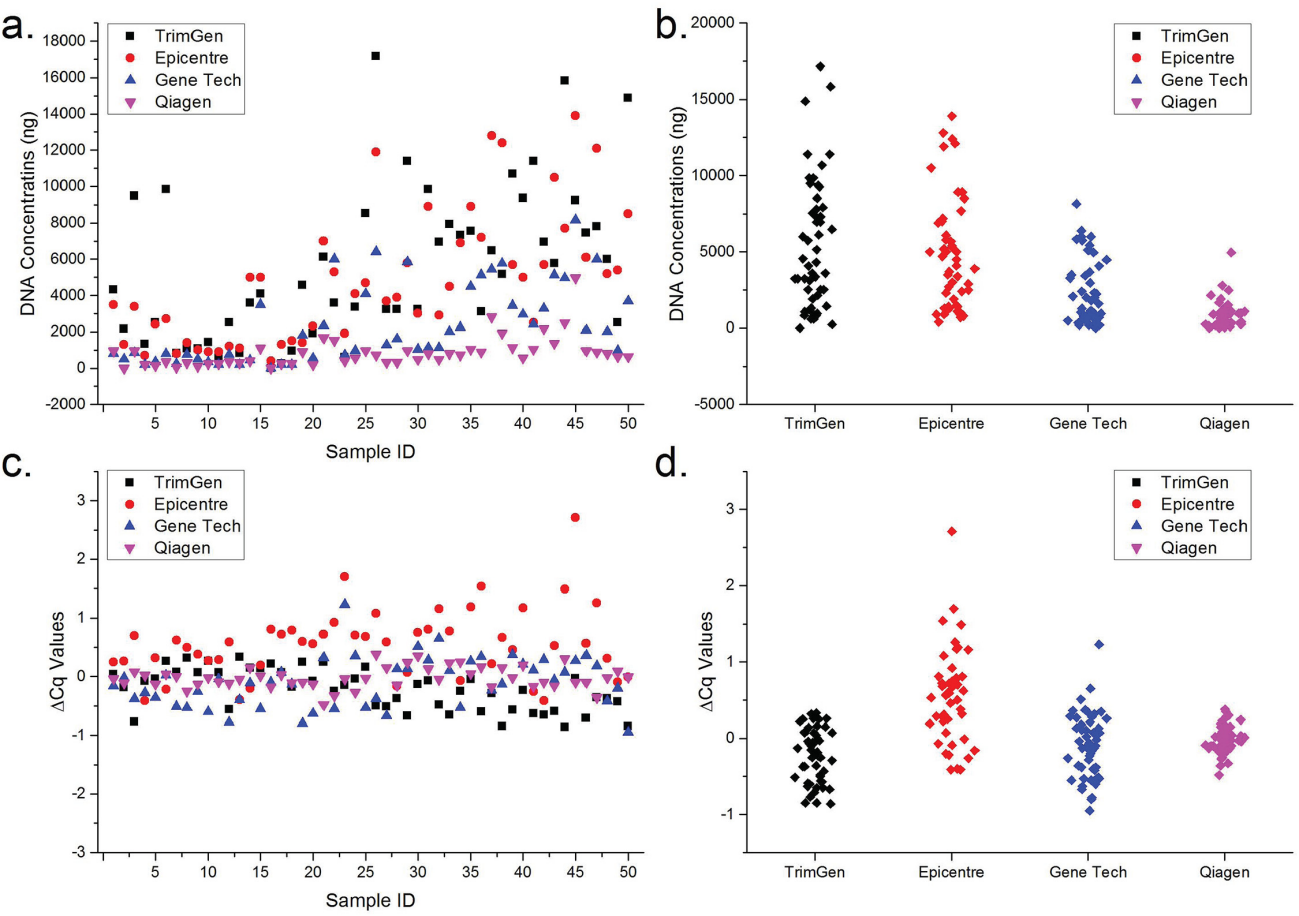
Comparison of the FFPE DNA extraction features associated with various commercially available kits. Panel a and b show the plot scatter and column scatter graphs generated based on the quantity of DNA isolated using four commercial kits, respectively. Panel c and d show the plot scatter and column scatter graphs of Δ*C*_q_ between FFPE and reference samples, respectively.

The characteristics of DNA extracted from FFPE sections can affect the accuracy associated with results generated from genotyping assays (including the real-time WTB-PCR assay). Although various commercially available kits for FFPE DNA extraction are available, it is necessary to consider both the quantity (i.e. amount of extracted DNA) and quality (i.e. amplifiable DNA without PCR amplification inhibitors) of the extracted DNA when analyzing which kits are most optimal for extraction. Therefore, in the present study, a comparative analysis was initially performed in relation to both the quantity and quality of DNA extracted following the use of four commercially available common FFPE DNA extraction kits (Fig 2). For the 20 samples scraped from the glass slide, the average amount of extracted DNA was 2670 ng (95% CI: 1385 to 3955 ng), 1910 ng (95% CI: 1272 to 2584 ng), 660 ng (95% CI: 173 to 297 ng), and 365 ng (95% CI: 209 to 521 ng) for TrimGen, Epicentre, Gene Tech, and Qiagen kits, respectively (Fig 2a and 2b). For the 30 samples that had been directly stored in microcentrifuge tubes, the average amount of extracted DNA was 7424 ng (95% CI: 5931 to 8917 ng), 6803 ng (95% CI: 5564 to 8043 ng), 3419 ng (95% CI: 376 to 2649 ng), and 1165 ng (95% CI: 173 to 811 ng) for TrimGen, Epicentre, Gene Tech, and Qiagen kits, respectively (Fig 2a and 2b). One-way ANOVA statistical analysis showed that there were significant statistical differences between the two different sample storage methods (*p* = 0.000). Interestingly, the TrimGen and Epicentre kits were the most effective for isolation of DNA from FFPE sections for both sample types. And there was no significant statistical difference between them (*p* > 0.05). However, the TrimGen kit facilitated increased isolation of DNA when compared with the Epicentre Kit, based on the average amount of DNA isolated. The Qiagen kit resulted in the lowest DNA yields for both sample types.

To compare further the abilities of the extraction kits to remove chemical agents that might inhibit amplification efficiencies, reaction systems containing spiked porcine gDNA were used to monitor potential PCR amplification inhibition in the FFPE DNA solutions. Human gDNA solution that did not contain PCR amplification inhibitors was used as a reference DNA control in the analysis. In the presence of amplification inhibitor residue, the *Cq* values of FFPE samples should be larger than that of the reference sample. The Δ*Cq* values between FFPE samples and reference samples were used to determine the presence of amplification inhibitors. The results showed that the average Δ*C*_q_ were -0.23 (95% CI: -0.33 to -0.13), 0.55 (95% CI: 0.38 to 0.72), -0.10 (95% CI: -0.22 to 0.02) and -0.02 (95% CI:-0.07 to 0.03) for TrimGen, Epicentre, Gene Tech, and Qiagen kits, respectively (Fig 2c and 2d). The results generated showed that there were significant differences between each of the kits analyzed (*p* = 0.000). Of the tested samples, a total of 10 samples had a Δ*C*_q_ value greater than 1.0, indicating that there were residual PCR amplification inhibitors in the extracted DNA solutions. Of these 10 samples, nine samples (Mean: 1.48; Range: 1.08 to 2.71) were isolated using the Epicentre kit and one sample (1.23) was isolated using a Gene Tech kit. According to the amount of DNA isolated and the residual amplification inhibitors present after isolation, we concluded that the TrimGen kit was the most effective because it resulted in the generation of the highest DNA yield with no visible residual inhibitors. Therefore, DNA isolated from the TrimGen kit was used in subsequent experiments.

### Optimization of real-time WTB-PCR system

To understand the thermodynamic properties and suppression efficiencies of WTB on the amplification of *KRAS* WT-alleles, serial concentrations of WTB (5.0, 2.0, 1.0, and 0.5 μM) were used in the WTB-PCR. Serial concentrations of human gDNA containing known *KRAS* WT-alleles (i.e., 100 and 50 ng each 20 μl of reaction mixture) were used in both real-time systems with (WTB-PCR) and without (traditional PCR) WTB. Fifty-five thermal cycles were used for these amplification reactions. The results showed that the *C*_q_ values were > 40 for each reaction mixture containing the various WTB concentrations. The Δ*C*_q_ values associated with the traditional and WTB-PCR reactions were used to select the optimum WTB concentration (Table 2). According to the maximum Δ*C*_q_ values generated, the results showed that higher concentrations of WTB gave rise to higher amplification suppression efficiencies. However, there was no significant difference between the minimum Δ*C*_q_ values for the various WTB concentrations. Δ*C*_q_ values generated using 0.5 μM WTB were sufficient to distinguish between WT- and MT-alleles. Moreover, standard deviation (SD) and coefficient of variance (CV) values indicated that the reaction system using 0.5 μM WTB resulted in the greatest reproducibility (Table 2). Based on both financial and reproducibility considerations, 0.5 μM of WTB was considered as the optimal final concentration in the WTB-PCR system.

**Table 2:** Optimization of WTB concentration in real-time PCR.

	Δ*C*_q_ (100 ng WT gDNA)				Δ*C*_q_ (50 ng WT gDNA)			
	5 μM	2 μM	1 μM	0.5 μM	5 μM	2 μM	1 μM	0.5 μM
**Maximum Δ*C***_**q**_ ^**#**^	25.53	26.67	22.17	16.79	26.27	25.79	25.78	19.77
**Maximum Fold** *	4.85×10^7^	1.07×10^8^	2.69×10^7^	1.25×10^6^	8.09×10^7^	8.09×10^7^	5.76×10^7^	9.78×10^5^
**Minimum Δ*C***_**q**_ ^**#**^	15.43	12.71	14.64	14.08	16.79	15.36	14.30	17.40
**Minimum Fold** *	4.53×10^5^	6.70×10^3^	2.72×10^4^	2.12×10^4^	1.13×10^5^	4.23×10^4^	2.02×10^4^	1.73×10^5^
**Average**	19.82	19.47	17.88	14.95	20.60	20.30	19.03	18.30
**SD** ^**#**^	4.82	5.00	2.82	1.06	4.63	4.45	4.22	0.87
**CV** ^**#**^	24.31%	25.68%	15.76%	7.09%	22.49%	21.95%	22.18%	4.74%

^#^Calculated from nine samples and three batches of triplex reaction mixtures. The Δ*C*_q_ values were calculated after 55 cycles. If there were no available *C*_q_ values for certain samples, the number 55 was used to calculate the *C*_q_ values in order to get informative data.

*Refer to fold-suppression, which was calculated using the equation 2ΔCq.

**Fig 3:**
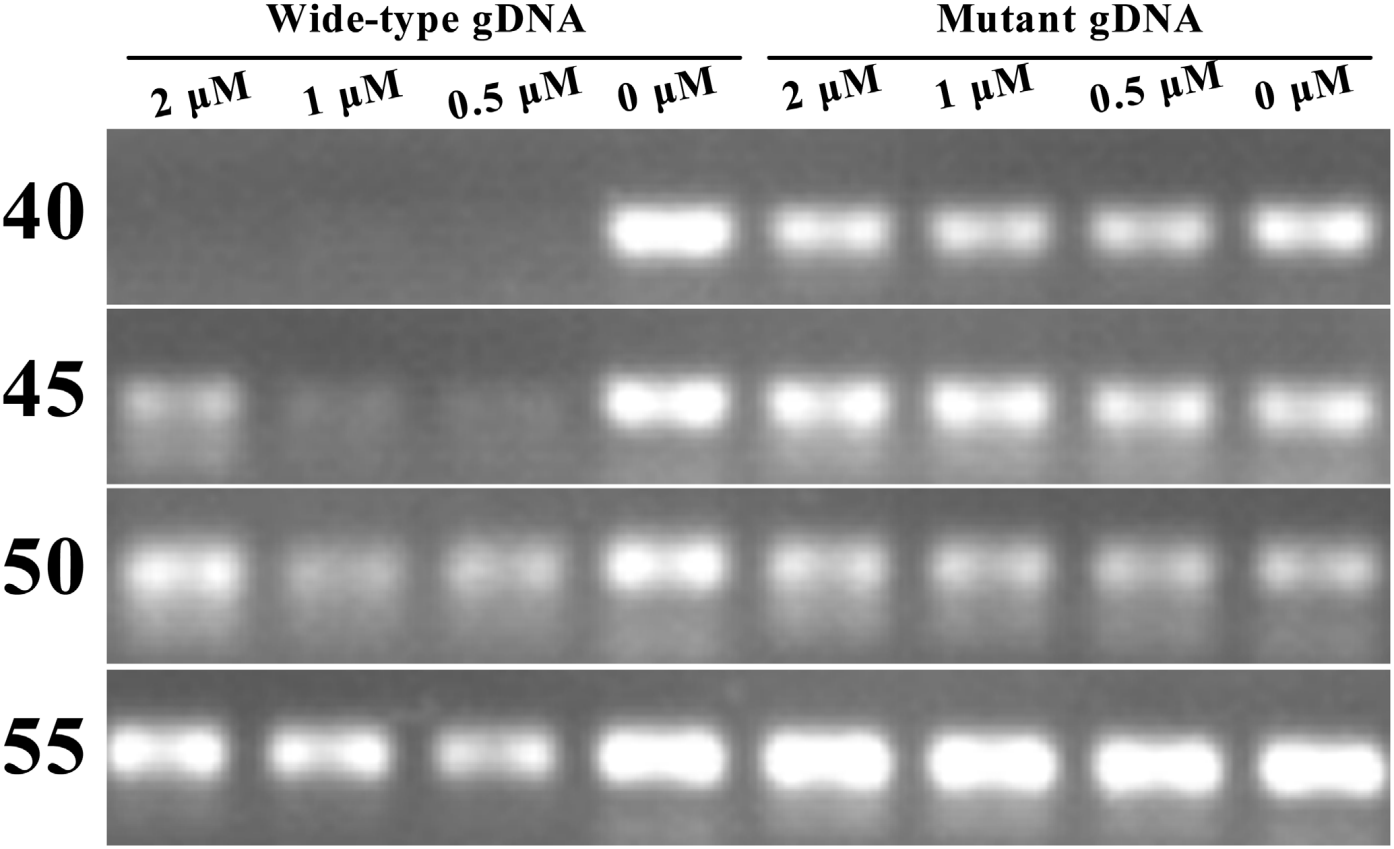
Gel images of amplicons generated after various cycle numbers. Reaction mixtures containing serial concentrations of WTB (2.0, 1.0, 0.5, and 0 μM) and homozygous WT- and MT-gDNA were performed in quadruplicate reaction tubes. At the specified cycle numbers indicated on the left hand side of image, one of the quadruplex tubes was taken out and placed on ice. When the reactions were complete, all of the samples were subjected to gel electrophoresis. The gDNA type and the WTB concentrations are labeled above the image.

The amplification mechanism associated with WTB-PCR should not facilitate amplification in reaction mixtures that contain WTB and WT-alleles. However, most of the samples used in this analysis had *C*_q_ values between 40 and 55 cycles, and these *C*_q_ values were independent of the WTB concentrations used. To explore these conflicting results, DNA sequencing was performed on the analyzed samples. The results showed that artificial adenosine substitutions occurred in the targeting region of the WTB oligonucleotide (data not shown). We speculate that this may have resulted from the lower-fidelity associated with *Taq* DNA polymerase that was used in this study. The polymerase used in this assay had no 3' to 5' exonuclease activity, and these artificial mutations may have occurred following the large number of thermal cycles that were used. As a result, the amplicons that were generated from reaction mixtures containing 50 ng of homozygous *KRAS* WT- or MT-gDNA (gDNA of AsPC-1 cell lines which had homozygous c.35G>A mutations) and incremental final WTB concentrations (2.0, 1.0, 0.5, and 0 μM) after specific numbers of thermal cycles (i.e., 40, 45, 50, and 55 cycles) were analyzed using agarose gel electrophoresis (Fig 3). The results showed that when WT-gDNA was used as the amplification template, visible amplicon bands appeared following gel electrophoresis after 45 amplification cycles had been used. In addition, reaction mixtures containing higher concentrations of WTB (e.g. 2 μM) tended to result in greater band intensities. At 55 cycles, there were no visible differences between traditional and WTB-PCR when reaction mixtures containing WT-gDNA were used (Fig 3). Based on these results, the optimal conditions that were used in the following experiments included the use of 40 amplification cycles and a reaction mixture containing 0.5 μM of WTB.

### Assessment of the sensitivity and quantitative capabilities of the real-time WTB-PCR system

To analyze the specificities of the WTB-PCR, MT-alleles were prepared using reverse primers containing specific mutated single nucleotides that mimicked each possible missense mutation at *KRAS* codons 12 and 13 (Table 1). All 12 possible MT-alleles were cloned to generate MT-QC plasmids. These mutations were confirmed following DNA sequencing (S1 Fig). The Δ*C*_q_ values generated following traditional and WTB-PCR reactions that used the 12 MT-QC plasmids as templates ranged from 0.33 to 0.77 (Table 3). This indicated that there was no significant suppression effects associated with amplification of the MT-alleles in the WTB PCR system (Table 3 and S2 Fig).

**Table 3:** Summary of the WTB suppression effects on the amplifications of 12 missense mutations at *KRAS* codons 12 and 13.

ReversePrimer ID	cDNAPositions	Protein Positions	Δ*C*_q_ Mean (*C*_qWTB_-*C*_qCTRL_)	Δ*C*_q_ SD (*C*_qWTB_-*C*_qCTRL_)
HQ-428	c.34G>C	p.G12R	0.63	0.30
HQ-429	c.34G>T	p.G12C	0.45	0.15
HQ-430	c.34G>A	p.G12S	0.33	0.13
HQ-431	c.35G>C	p.G12A	0.45	0.07
HQ-432	c.35G>T	p.G12V	0.43	0.16
HQ-433	c.35G>A	p.G12D	0.75	0.04
HQ-434	c.37G>C	p.G13R	0.40	0.02
HQ-435	c.37G>T	p.G13C	0.77	0.54
HQ-436	c.37G>A	p.G13S	0.34	0.18
HQ-437	c.38G>C	p.G13A	0.38	0.19
HQ-438	c.38G>T	p.G13V	0.48	0.49
HQ-439	c.38G>A	p.G13D	0.59	0.17

**Fig 4:**
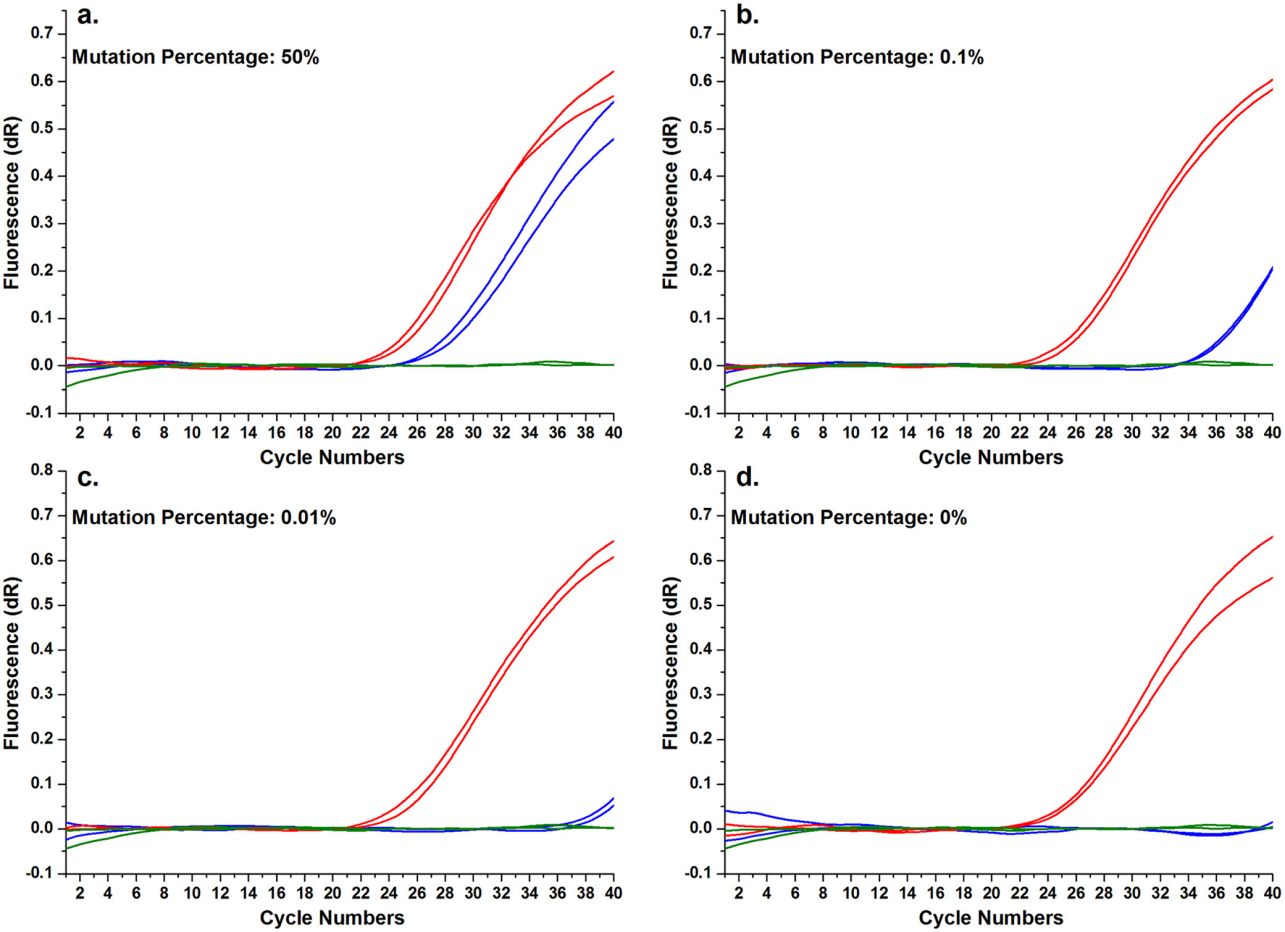
Enhanced sensitivity of the real-time WTB-PCR system. Panel a to d show the amplification curves of real-time PCR systems with (indicated by blue lines) and without (indicated by red lines) WTB and specified concentrations of MT-alleles (indicated in each panel), in which the olive curves indicated NTC reactions. In a 20 μl reaction mixture, increasing quantities of AsPC-1 gDNA (containing a homozygous mutation at c.35G>A; 5, 10, 50, 500, 5,000, 12,500, 25,000, 50,000 pg) were spiked into samples containing WT-gDNA, giving a total of 50 ng of gDNA. These were used to prepare templates containing increasing mutant quantities (i.e. 0.01, 0.02, 0.1, 1, 10, 25, 50, 100%). This image shows amplification curves following the use of specific mutant concentrations in the template DNA. The amplification curves associated with all 12 missense mutations and the associated mutant quantities used as templates are available in S2 Fig. The *C*_q_ values of reaction mixtures without WTB were consistently lower than those of reaction mixtures with WTB, suggesting that the WTB-PCR is a sensitive assay.

To determine the sensitivity of the real-time WTB-PCR system, a concentration gradient of mutant template was prepared by mixing increasing concentrations of gDNA from AsPC-1 cell lines containing homozygous *KRAS* MT-alleles (i.e. c.35G>A; p.G12D) with human WT-gDNA (Fig 4 and S3 Fig). The data generated showed that traditional PCR always resulted in consistent *C*_q_ values despite the differences in the mutated template concentrations associated with the *KRAS* MT-alleles. These results indicated that both WT- and MT-alleles could be equivalently amplified. Therefore, the ***C***_**q**_ values associated with traditional PCR can be used to indicate the total quantity of input DNA [36]. However, when WTB-PCR was utilized, the *C*_q_ values progressively increased proportionally with the quantity of *KRAS* mutation template, indicating that the amplification of *KRAS* WT-alleles was efficiently inhibited or eliminated in accordance with the principles of WTB-PCR (Figs 1b and 4, S1 Fig). Comparative analysis of both sets of amplification curves and *C*_q_ values for each sample in traditional and WTB-PCR indicated that the current real-time WTB-PCR facilitates the detection of *KRAS* MT-alleles with sensitivity levels as low as 0.01% (or a 1:10,000 ratio) (Fig 4 and S3 Fig).

**Fig 5:**
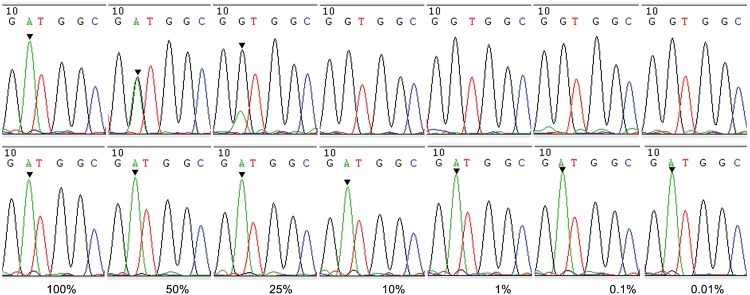
Sequencing results of WTB-PCR products from DNA samples containing various percentages of mutated *KRAS* alleles. Sequencing chromatographs are shown for the products from the real-time traditional PCR (top row) and WTB-PCR (bottom row) reactions in Fig 4 and S3 Fig. The numbers at the bottom of each segment indicate the percentages of mutated *KRAS* c.35G>A allele in the PCR reaction mixtures. The arrows indicate the position of the G to A mutation of the *KRAS* c.35G>A allele.

**Fig 6:**
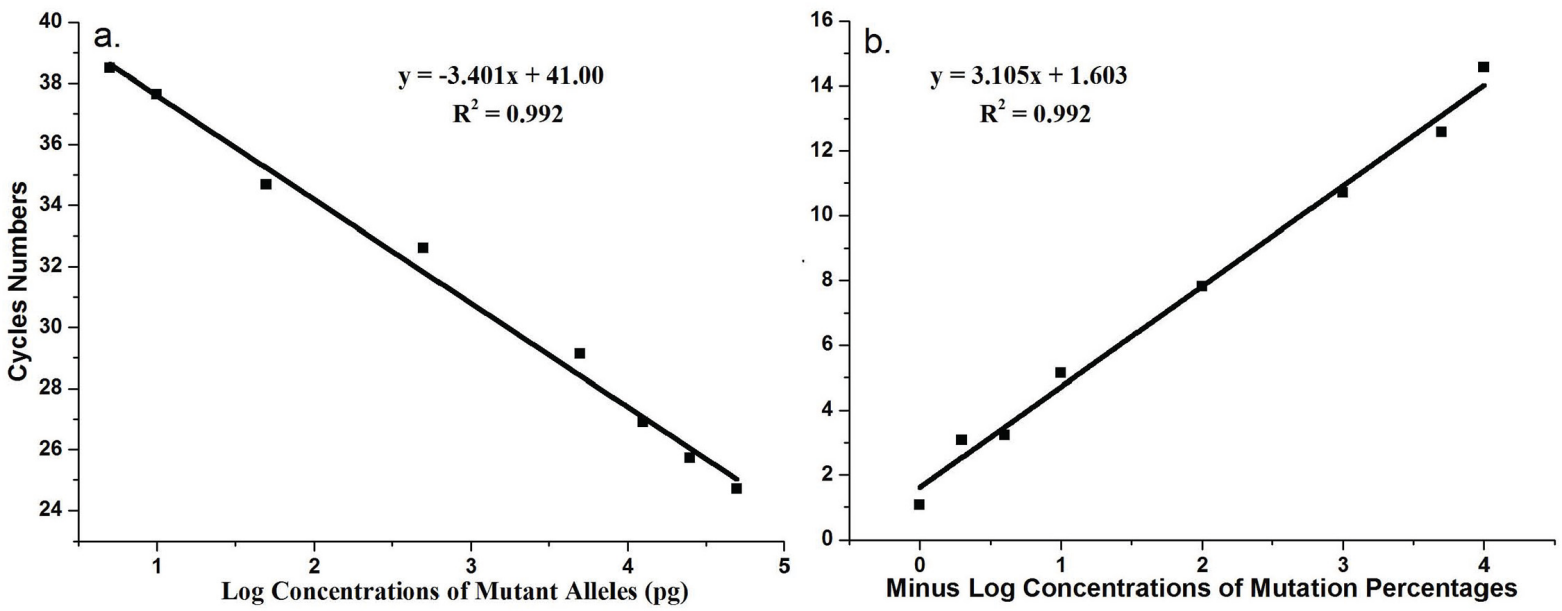
Quantitative curves of real-time WTB-qPCR. a. A standard curve was generated by plotting the average *C*_q_ values from real-time WTB-PCR against the log concentrations of mutant *KRAS* alleles (i.e. 5, 10, 50, 500, 5,000, 12,500, 25,000, 50,000 pg). The total amount of template was 50 ng of genomic DNA containing increasing levels of the mutated c.35G>A allele (i.e. 0.01, 0.02, 0.1, 1, 10, 25, 50, and 100%). The average *C*_q_ values are shown in Fig 4 and S3 Fig. These were automatically determined with MxPro Software. The amplification efficiency of real-time WTB-PCR was 96.8% (slope, -3.401; *R* ^2^ = 0.992). b. A standard curve was generated by plotting the average Δ*C*_q_ values between real-time traditional PCR and WTB-PCR against minus log percentage concentrations of mutant c.35G>A alleles (i.e. 0.01, 0.02, 0.1, 1, 10, 25, 50, and 100%) in 50 ng of genomic DNA. The standard curves from panel b were used to calculate the percentage of *KRAS* mutant alleles in heterozygous genomic DNA.

To confirm these results, DNA sequencing was performed to analyze the PCR products associated with both traditional and WTB-PCR. The sequencing results from the traditional PCR amplicons were equivalent to that generated following routine Sanger sequencing (Fig 1a). The traditional PCR approach allowed for the detection of approximately 25% *KRAS* MT-alleles only (Fig 5). This mutation identification sensitivity was consistent with that of Sanger sequencing, as identified in previous reports [33,37,38]. However, for WTB-PCR, the amplification of *KRAS* WT-alleles was completely inhibited until the proportion of *KRAS* MT-alleles in the template was as low as 0.01% (Fig 5). Based on the amplification curves and sequencing chromatographs (Figs 4 and 5), we concluded that the current WTB-PCR was capable of detecting a single copy of *KRAS* MT-allele in the presence of a 10,000-fold excess of WT-alleles with an amplification efficiency of 96.8% (R^2^ = 0.992) (Fig 6a). Furthermore, a plot of the Δ*C*_q_ between the traditional PCR and WTB-PCR systems suggest a linear association with the quantity of template containing the *KRAS* mutation (R^2^ = 0.992), indicating that the amount of MT-alleles in a given sample could be quantified by calculating the Δ*C*_q_ between the two PCR systems (Fig 6b).

### Applications of real-time WTB-PCR to test FFPE specimens from clinical mCRC patients

**Fig 7:**
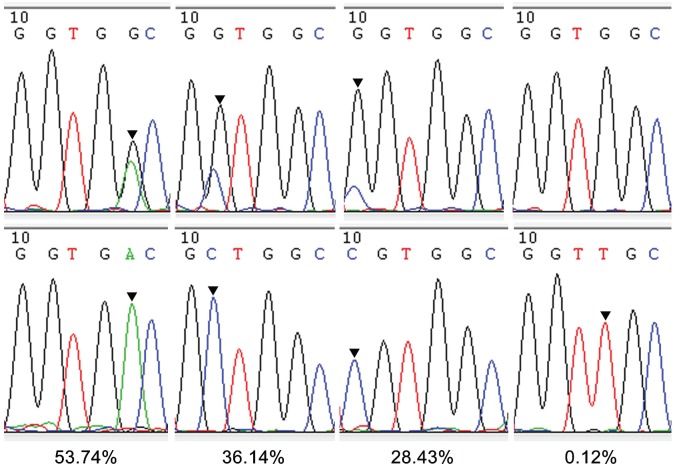
Examples of results obtained from clinical samples. Sequencing chromatographs are shown for the products from the real-time traditional PCR (top row) and WTB-PCR (bottom row) for three clinical FFPE samples. The numbers at the bottom of each segment indicate the percentages of mutated *KRAS* allele in the PCR reaction mixtures. The arrows indicate the position of each mutated nucleotide in the *KRAS* allele.

Following analysis of the 50 FFPE samples, only 49 samples resulted in positive *KRAS* amplification using traditional PCR. However, only 30 samples showed positive amplification when WTB-PCR was used. Furthermore, sequencing of the products generated following WTB-PCR showed that all 30 positively amplified samples were predominately composed of *KRAS* MT-alleles (Fig 7; Table 4). In contrast, only 19 of the 30 mutated samples could be detected following sequencing analysis of the traditional PCR products. Therefore, the WTB-PCR system resulted in a much higher positive percentage (i.e., 61.8%; 30/49) in relation to the presence of mutated samples that were detected following sequencing analysis when compared with the percentage of mutated *KRAS* detected following traditional PCR (i.e. 38.8%; 19/49). According to the standard curve presented in Fig 6b, the Δ*C*_q_ between traditional and WTB-PCR systems for these 19 samples suggested a mutant percentage of greater than 25% (range: 53.74 to 24.43%). However, the 11 samples that were not analyzed using traditional PCR gave rise to a *KRAS* mutant percentage range between 21.25% and 0.12%. Interestingly, when compared with the preliminary WTB-PCR system using 55 cycles (S1 Table), the DNA sequencing results of the optimized WTB-PCR (using only 40 cycles) showed no artificial mutant nucleotides. Moreover, of the 11 mutated samples that were not confirmed by traditional PCR, only four samples showed G>A mutations (one sample contained the c.34G>A mutation and three samples contained the c.35G>A mutation). Interestingly, the peak associated with the mutant base A was much higher than that of the wild-type base G in these four samples (S1 Table). Moreover, all of the mutated bases associated with the other seven samples were present in the amplicons generated following the preliminary 55-cycle system. The chromatograph peak height associated with these mutations was always higher than that of both the wild-type base G and the artificial A mutation (S1 Table). These results indicated that maintaining a low thermal cycle number was essential in avoiding transcription errors introduced by *Taq* DNA polymerase lacking 3' to 5' exonuclease activities.

**Table 4:** Summary of *KRAS* mutation analysis of 49 clinical samples.

		Mutated Cases	
cDNA Positions	Protein Positions	PCR	WTB-PCR
c.34G>C	p.G12R	1	1
c.34G>T	p.G12C	1	1
c.34G>A	p.G12S	ND	1
c.35G>C	p.G12A	2	5
c.35G>T	p.G12V	1	3
c.35G>A	p.G12D	6	9
c.37G>C	p.G13R	ND	1
c.37G>T	p.G13C	1	2
c.37G>A	p.G13S	ND	ND
c.38G>C	p.G13A	ND	ND
c.38G>T	p.G13V	ND	ND
c.38G>A	p.G13D	7	7
**TOTAL**		**19**	**30**
**Percent**		**38.8%**	**61.2%**

## Discussion

*KRAS* is the most commonly mutated gene in the RAS/RAF/MAPK pathway, with approximately 35% to 45% of mCRC patients harboring *KRAS* mutations [39]. KRAS mutations can result in constitutive activation of the RAS/RAF/MAPK pathway, and therefore confer resistance to EGFR-antibody mediated therapies used in the treatment of mCRC [2,39–43]. Up to 90% of the mutations that result in activation of the *KRAS* gene are detected in codons 12 (82–87%) and 13 (13–18%) [2,39–43]. The most common types of *KRAS* mutations associated with CRC are G-to-A transitions and G-to-T transversions [39]. Moreover, a study showed concordance rates of 96.4% in *KRAS* codons 12 and 13 mutations, and pooled results from several studies of paired primary and hepatic metastatic tumors showed concordance rates of 91% to 92% [2] The presence of KRAS mutations was determined to be predictive of resistance to anti-EGFR therapy.

The large number of FFPE samples used as part of this analysis provided an extremely valuable pathologically disease-specific bank of material that could be used in molecular biological research pertaining to routine clinical diagnoses. The quantity and quality of DNA isolated following various FFPE DNA extraction methods using in-house reagents and commercially available kits are imperative for various molecular analysis methods. Sufficient quantities of amplifiable DNA are required to attain reliable results, ensuring elimination of false negative results resulting from the presence of residual PCR amplification inhibitors in extracted DNA solutions [37,44]. Indeed, although various commercially available kits can be used to efficiently extract DNA from FFPE sections, these kits are not directly intended for diagnostic purposes. Until now, there has been relatively limited research describing the properties of commercial kits in this area, and any data that was generated suggested that it was important to select kits that were optimal for specific applications [45–47]. In the present study, both the quantity and quality of DNA generated using four commonly used commercial kits were systematically evaluated to initially determine which one facilitated the generation of the most consistent yields of amplifiable DNA. This was assessed in order to determine which kit provided sufficient quantities of amplifiable DNA for various genotyping assays including the real-time WTB-PCR assay developed as part of this study.

Of the four commercial kits that were evaluated in this study, two of them required column extraction methodology, where the extraction procedure was comparatively complex. The other two kits were comparatively simple to use, with non-binding and non-elution methods required to extract DNA from the sections. Compared with column- (e.g. Qiagen and Gene Tech kits) or magnetic bead-based methods, non-binding and non-elution methods (e.g. TrimGen and Epicentre kits) facilitated more efficient DNA extractions from FFPE sections (*p* < 0.05%).

In order to evaluate the presence of potential residual PCR amplification inhibitors in the extracted DNA solutions, we used cross-species non-homozygous nucleic acid materials as internal positive control (IPC) templates in PCR-based reactions [48]. The Δ*C*_q_ values between the spiked mixture (containing both IPC and FFPE DNA solutions) and reference mixture (containing only IPC) facilitated the analysis that determined the presence of inhibitors [48]. Our results suggest that the simple extraction procedure might facilitate the transfer of inhibitors in the DNA extract. For example, 18% (9/50) of the samples extracted using the Epicentre kits contained PCR inhibitors. However, only 2% (1/50) of the samples extracted using the column method (i.e., Gene Tech) showed residual PCR inhibitors. Moreover, the residual inhibitors could not be completely eliminated even when additional commercial kits such as TF Filter Tips (TrimGen), SigmaSpin Sequencing Reaction Clean-Up (Sigma-Aldrich), and QIAquick PCR Purification Kit (Qiagen) were used to further purify the extracted DNA (data not shown). Interestingly, the kit that generated the greatest amount of DNA with no residual inhibitor was the TrimGen kit. This is consistent with previous reports [45–47]. Therefore, based on considerations pertaining to the quality and quantity of extracted DNA, the data suggests the TrimGen kits are the optimal choice for the clinical applications.

Because of intratumor heterogeneity, tumor cells containing mutated *KRAS* alleles frequently present as subclones in mCRC. Following the development of highly sensitive methods to detect minority *KRAS* subclone mutants in the presence of large excesses of wild-type *KRAS* subclones, research is currently being conducted to determine whether methods with greater sensitivity could be used to exclude a greater number of mCRC patients who might not benefit from anti-EGFR antibody treatment [9–12,35,49]. Compared with Sanger sequencing, which demonstrates limited mutational detection sensitivity (approximately 20% to 25%), moderately sensitive methods (sensitivities ranging between 1% and 10%) including pyrosequencing, matrix-assisted laser desorption/ionization time-of-flight mass spectrometry (MALDI-TOF MS), SNaPshot, PCR-ligase chain reaction (LCR), PCR-RFLP, and ARMS-scorpion assay (ARMS/S) are likely to enhance the identification of mCRC patients who will not respond to anti-EGFR treatment [9–12,35,49]. These results indicate that it is necessary to use at minimum a moderately sensitive method to detect mutated *KRAS* genes to exclude mCRC patients who might not benefit from anti-EGFR antibody treatment. Moreover, because of limited sensitivity, Sanger sequencing should not be used to evaluate *KRAS* mutations when identifying mCRC patients who might benefit from anti-EGFR antibody treatment in clinical oncology. In a recent publication, results generated by Laurent-Puig and colleagues suggested that mCRC patients harboring 1% *KRAS* mutated subclones (detected using ultra-high sensitivity picodroplet dPCR, with 0.0005% sensitivity) could benefit from anti-EGFR treatment [49]. The reason for these results may be that most of the tumor cells were wild-type *KRAS* subclones that could respond to anti-EGFR antibody treatment in the early stages of treatment. As a result, mCRC patients with low levels of *KRAS* mutated subclones could benefit from anti-EGFR treatment compared with patients who have higher *KRAS* mutated subclones. However, as indicated by Laurent-Puig and others [49–52], even low levels of mutated *KRAS* subclones might be sufficient to allow the development of resistance due to the fact that pre-existing mutated subclones might selectively proliferate in the presence of anti-EGFR antibodies. Moreover, considering the likely lower levels (as low as 0.037%) of *KRAS* mutated alleles present in the plasma or serum circulating cell-free DNA (ccfDNA) [25,27,28], it was necessary to develop *KRAS* genotyping methods that have mutation detection sensitivities of at least 0.037% or higher (the real-time WTB-PCR has a detection sensitivity of 0.01%) in order to non-invasively detect and monitor *KRAS* mutations in mCRC patients during anti-EGFR treatment. Therefore, as suggested by Laurent-Puig and others [9–12,35,49], it was necessary to use higher sensitivity methods to detect low-abundance mutated *KRAS* genes to facilitate determination of patients that might benefit from anti-EGFR antibody treatment.

Of the various methods used to detect low-abundance mutated genes, clamp PCR mediated by WTB oligonucleotides such as PNA [53–56], LNA [31,32], and LNA/DNA chimeras [33,38], facilitate the discrimination of single base mismatches, and have shown more optimal performance in the detection of mutant alleles with sensitivities of up to 0.01%. Two common strategies are used in WTB mediated clamp PCR; ‘primer exclusion’ and ‘elongation arrest’ [53,54]. Using the primer exclusion strategy (Fig 2b), the targeted sites of the WTB oligonucleotide partially overlap with the amplification primers [31,34,38,54,55]. In the elongation arrest strategy, the targeted sites of the WTB oligonucleotide are located at the 3'-flanking region of the primers [32–34,53,54]. In both cases, the complementary binding between WTB and wild-type alleles inhibits or eliminates the amplification of wild-type alleles, thereby selectively amplifying mutant alleles. This occurs because single base mismatches can potentially disrupt the duplex between WTB and mutant alleles [31–34,38,53–55]. Compared with other low-abundance mutation analyses using allele-specific primers or probes, the major advantage of WTB-mediated clamp PCR is that it can be used to detect single base variations in a stretch containing several base pairs that are complementary to the WTB sequence. Again, this is because a single base mismatch can disrupt the duplex between WTB and its targeted sequences. Using LNA/DNA chimeras as WTB and following the primer exclusion strategy, the real-time WTB-PCR used in this study can simultaneously detect all 12 possible missense mutations at codons 12 and 13 of *KRAS* genes in a single closed tube. Based on the advantages associated with our real-time PCR assay, similar strategies could be used to sensitively detect low-abundance mutations in other codons (e.g. codons 59, 6, 117, and 146) of *KRAS* and other oncogenes including NRAS (e.g. codons 12, 13, 59, 6, 117, and 146), BRAF (e.g. codons 594. 596, and 600), and PIK3CA (e.g. exon 9 and 20). This strategy would facilitate the exclusion of mCRC patients harboring the wild-type *KRAS* gene sequence at codons 12 and 13 in relation to anti-EGFR antibody treatment [57–60]. For example, WTB targeting the wild-type sequence of the *NRAS* gene at codons 12 and 13 could be used to sensitively detect all 12 possible *NRAS* mutated alleles in a single tube. This further suggests the use of the WTB-mediated PCR assay in the detection of low-abundance mutant alleles to exclude mCRC patients who might not benefit from anti-EGFR antibody treatment.

In previous clamp PCR assays that targeted *KRAS* genes, PNA was predominantly used as the WTB oligonucleotide with primer exclusion or elongation strategies. This strategy was combined with other technologies, such as HRMA [55], mutant-specific hybridization probes or detection probes [61]. The associated sensitivity of these hybrid strategies ranged from 1% to 0.01% [55,56,61,62]. Compared with previous clamp PCR assays that targeted *KRAS* genes where PNA was used as the WTB oligonucleotide, our group have developed LNA/DNA chimeras that facilitate the clamp PCR [33]. Because of the mismatch discrimination abilities of LNA/DNA chimeras [63], LNA substitutes were mainly modified at the potential mutation sites of the *KRAS* gene. As a result, the sense strand of the *KRAS* gene was targeted to avoid the decreased discrimination associated with G-T mismatches [33]. Our results further confirmed that previously published LNA-DNA chimeras serve as blockers that enable determination of single base mismatches. Our strategy has similar sensitivity levels (i.e. 0.01%) with previous clamp PCR assays that used PNA or LNA/DNA chimeras to target *KRAS* codons 12 and 13 [33,55,56,61,62]. However, when compared with previous LAN/DNA-mediated clamp PCR assays that were based on the elongation arrest strategy, the current method uses a primer exclusion strategy with additional hydrolysis probes to allow for the real-time monitoring of possible mutations. This assay also allows simultaneous parallel monitoring of reaction mixtures with and without WTB to quantitate mutant presence associated with the *KRAS* gene. In comparison with the clamp PCR assay that targeted *KRAS*, the major advantages associated with our method include the following: the amplicon size (118 bp) associated with *KRAS* amplification is more optimally amplified using this method; the proportion of mutant alleles in the sample can be quantitated using Δ*C*_q_ values between reaction mixtures with and without the WTB oligonucleotide; the LNA/DNA chimera is more cost effective than PNA blockers. These advantages offer this method as a more attractive assay for clinical applications.

In preliminary experiments, as many as 55 cycles were used to generate a sufficient quantity of template for Sanger sequencing. However, we observed that higher numbers of cycles resulted in the artificial introduction of mutant nucleotides in amplicons (S1 Table). In the present study, in order to increase the mismatch discrimination ability of the assay, the sequences of *KRAS* codons 12 and 13 were positioned in the center of the LNA/DNA chimeras. Previous studies had indicated that mismatches in the center of DNA/LNA or DNA/PNA duplex created greater instability than terminally placed mismatches [63,64]. The sequencing chromatograph associated with amplicons following 55 amplification cycles indicated that most of the artificial mismatches were located in the central position of the LNA/DNA chimeras and the complementary positions associated with LNA substitutes (S1 Table). Moreover, up to five mismatches were observed in the central positions that corresponded to the WTB oligonucleotide sequence. Interestingly, we observed an overrepresentation of G>A errors in the amplicons amplified from the WT-template with the heterozygous templates giving rise to much lower mutant percentages than the MT-template. Each of these artificial mismatch patterns were similar to those observed in previous publications where *Taq* DNA polymerase was used in PNA mediated clamping PCR reactions [34]. Moreover, similar to previous publications [34], the WT-peaks were always higher than those resulting from artificially introduced mutations. As indicated in previous publications, the artificial mutations were introduced because of errors in *Taq* DNA polymerase mediated replication. In addition, any of these errors that led to mismatches between WTB and WT-template were enriched during the PCR due to weaker WTB-clamping when compared with wild-type template [34]. Although several studies have reported that the base substitution specificity associated with *Taq* DNA polymerase mediated replication indicates a tendency toward T>C errors [65,66], other studies relating to PCR mutagenesis have shown that the mutational specificity of *Taq* polymerase can be altered by using different conditions for the PCR [67]. Indeed, our results and results generated in previous studies indicate that *Taq* DNA polymerase has a wider mutational specificity than Platinum, HotGoldStar, and *Ex Taq* DNA polymerases [34]. All of these studies indicate that the substitution specificity associated with *Taq* polymerase has a tendency toward C>T in oligonucleotide-mediated clamp PCR [34].

Although high-fidelity DNA polymerases, such as Phusion HS DNA polymerase or iProof High-Fidelity DNA polymerase, can be used to reduce or eliminate replication errors, thereby decreasing the introduction of artificial mutations in clamp PCR reactions [34,38], the use of high-fidelity DNA polymerases has limitations. For instance, when high-fidelity DNA polymerases are used, DNA-specific binding fluorescent dyes such as EvaGreen (Biotium, Hayward, CA, USA) or SYBR Green I are required to negate deficiencies associated with the 5'-to-3' exonuclease activities of these polymerases [68–70]. As a result, other real-time PCR strategies, such as the use of hydrolysis probes, hybridization probes, or molecular beacons cannot be used to sequence amplicons, thereby making it difficult to analyze simultaneously the internal control fragment. Although as many as 75 PCR amplification cycles have been used in previous publications [55], our results indicate that too many thermal cycles might introduce artificial mutations because of the lower fidelity associated with *Taq* DNA polymerase. The results of this analysis indicated that lowering the number of thermal cycles to 40 cycles was enough to limit the generation of false extension products introduced by DNA polymerase lacking high-fidelity properties.

Of the 12 possible mutations at *KRAS* codons 12 and 13, the most frequent mutations are the six missense mutations associated with codon 12 (p.G12R, p.G12C, p.G12S, p.G12A, p.G12V, and p.G12D) and the single mutation at codon 13 (i.e., p.G13D) [39]. In the present study, 90% (27/30) of the samples tested contained missense mutations at the latter positions, with only 25.9% (7/27) occurring at codon 13 (Table 4). Although commercially available kits targeting *KRAS* mutations have focused on these seven common mutation sites, there is a need to further develop methods that target other mutations sites, such as the two missense mutation sites (i.e., p.G13R and p.G13C) at codon 13 (that occur at a frequency of at least 10%; Table 4).

According to recent publications, circulating tumor DNA (ctDNA) analysis is non-invasive and enables day-to-day patient follow-up and monitoring in relation to treatment response. For instance, monitoring of ctDNA *KRAS* mutations in mCRC patients allows us select patients prior to initiation of anti-EGFR treatment or to monitor responses during anti-EGFR treatment [13,22,25,27,28,30]. For example, Diaz and colleagues highlighted that 38% (9/24) of patients presenting with a tumor who were initially classified as wild-type *KRAS* developed detectable mutations in *KRAS* in serum analyzed during or after treatment [51]. Moreover, ctDNA provides a more optimal diagnosis of tumor heterogeneity than a biopsy taken from a primary tumor. However, because levels as low as 0.037% mutant KRAS alleles can be present in serum or plasma circulating cell-free DNA (ccfDNA) in mCRC patients [25,27,28], it was necessary to develop a highly sensitive mutation detection assay (such as IntPlex and picodroplet dPCR) to detect mutant *KRAS* alleles in ccfDNA, to avoid false negative results [13,22,25,27,28,30,49]. For example, at least 15% of mutant samples screened using the TaqMan allelic discrimination assays or NGS methods failed detection when compared with picodroplet dPCR and IntPlex [25,49]. Using allele-specific blockers and primers with low melting temperatures (Tm), IntPlex, which was specifically designed to analyze ctDNA, is capable of detecting mutant *KRAS* alleles with ultra-sensitivity ranging from 0.004% to 0.012% [25,27,28]. However, because this method requires the use of allele-specific primers, detection of each possible mutation associated with *KRAS* at codons 12 and 13 requires a separate reaction tube [25,27,28]. Compared with IntPlex [25,27,28], picodroplet dPCR can precisely quantify mutated *KRAS* alleles in the presence of a 200,000-fold excess of unmutated *KRAS* alleles (i.e. 0.0005%). Additionally, this method can facilitate the detection of the seven most prevalent *KRAS* mutations using only two reactions i.e. using one 4-plex in conjunction with one 5-plex assay [13,22,30]. Additionally, the sensitivity associated with picodroplet dPCR might be further increased. The associated sensitivity is limited only by the number of picodroplets generated and analyzed by the accompanying instrument and software [13,22,30]. However, at present, picodroplet dPCR is still expensive, complex and time-consuming [13,22,29,30]. Moreover, it requires complex optimization to perform multiplex assays due to limitations associated with instrument fluorescence channels [29]. When compared with the IntPlex and picodroplet dPCR methods, the sensitivity of our real-time WTB-PCR assay (≤ 0.01%) was approximately 40- and 500-fold lower than that of IntPlex (≤ 0.004%) and picodroplet dPCR (≤ 0.005), respectively. However, the sensitivity of our real-time WTB-PCR assay is likely to have potential applications in ctDNA analysis with the associated sensitivity greater than that required for known mutant KRAS alleles in ccfDNA (i.e. up to 0.037%). In comparison with IntPlex and picodroplet dPCR, the major advantages associated with our real-time WTB-PCR assay are that it facilitates detection of all 12 possible mutations of the *KRAS* gene at codons 12 and 13 in a single tube in approximately 60 minutes. Moreover, this assay only requires the use of a real-time PCR instrument, which is commonly available in most clinical laboratories.

## Conclusion

A real-time WTB-PCR method was developed to assess all 12 possible missense mutations of the *KRAS* gene at codons 12 and 13 with as much as 0.01% sensitivity in a single close tube. Using human gDNA containing serial dilutions of the *KRAS* MT-allele, we calculated the Δ*C*_q_ between real-time traditional PCR and WTB-PCR, and then generated a calibration standard that was applied to determine the presence of the MT-allele in mCRC. This approach allows quantitative analysis of mutated alleles in heterozygous clinical tumor samples.

## Supporting Information

S1 Fig Summary of the quality control (QC) plasmid used in this study. Panel a to c shows the sequencing chromatograph of the WT-QC plasmid, the sequence alignment results between WT-alleles and 12 MT-QC plasmids, and the sequencing chromatograph of the 12 MT-QC plasmids, respectively.
(PDF)

S2 Fig Amplification curves of real-time PCR with and without WTB targeting each of the 12 missense mutations at *KRAS* codon 12 and 13. Pane a to l show the amplification curves of real-time PCR with and without WTB targeting the 12 MT-QC plasmids, respectively.
(PDF)

S3 Fig Sensitivities of real-time WTB-PCR. Panel a to i show the amplification curves of samples containing specific c.35G>A allele mutation percentages of 100%, 50%, 25%, 10%. 1%, 0.1%, 0.02%, 0.01%, 0%, respectively.
(PDF)

S1 Table Sequencing results of amplicons generated after 55 amplification thermal cycles. All targeted sequences overlapping with WTB are listed. And only mutated bases that were observed following sequencing results are shown.
(PDF)
