# Supplementary material for: Single-Tubed Wild-Type Blocking Quantitative PCR Detection Assay for the Sensitive Detection of Codon 12 and 13 KRAS Mutations
Source: PLoS One. 2015 Dec 23;10(12):e0145698. doi: 10.1371/journal.pone.0145698 (PMC4689371; doi:10.1371/journal.pone.0145698)
Supplement: S1 Fig — Panel a to c shows the sequencing chromatograph of the WT-QC plasmid, the sequence alignment results between WT-alleles and 12 MT-QC plasmids, and the sequencing chromatograph of the 12 MT-QC plasmids, respectively. (PDF) [file pone.0145698.s001.pdf]

Figure S1. Summary of the quality control (QC) plasmid used in this study

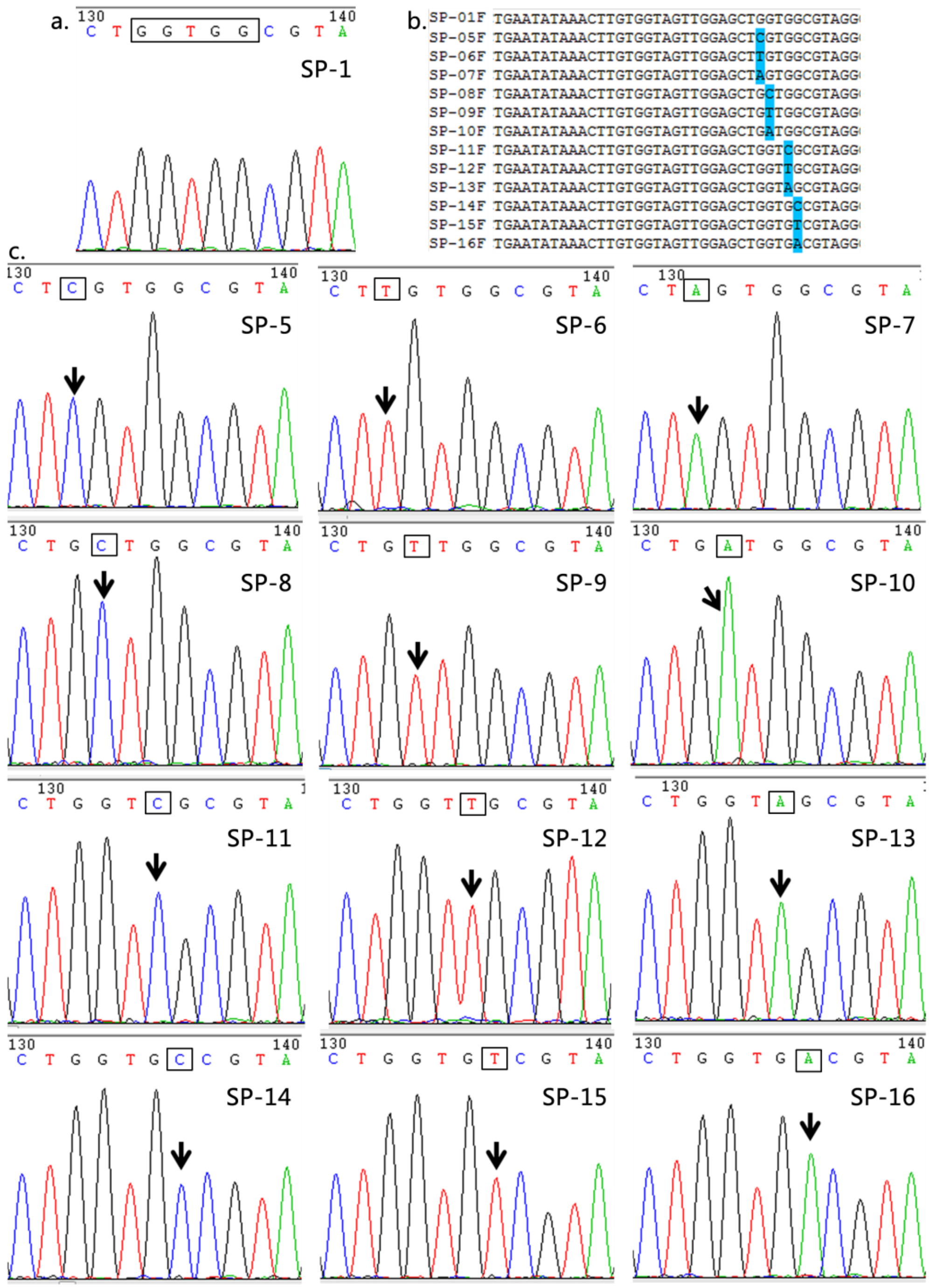

Panel a shows the sequencing chromatograph of the WT-QC plasmid (termed SP-1 in the present study), in which the rectangle box in panel A shows the nine bases in wild-type *KRAS* codons 12 and 13. Panel B shows the sequence alignment results between WT-alleles and 12 MT-QC plasmids (termed SP-5 to -16). The blue color indicates the mutated bases in each QC plasmid compared with WT-alleles. Panel C showed the sequencing chromatograph of the 12 MT-QC plasmids, where the rectangles and arrows indicate the mutated bases and the corresponding chromatograph peaks, respectively.
