# Supplementary material for: Single-Tubed Wild-Type Blocking Quantitative PCR Detection Assay for the Sensitive Detection of Codon 12 and 13 KRAS Mutations
Source: PLoS One. 2015 Dec 23;10(12):e0145698. doi: 10.1371/journal.pone.0145698 (PMC4689371; doi:10.1371/journal.pone.0145698)
Supplement: S2 Fig — Pane a to l show the amplification curves of real-time PCR with and without WTB targeting the 12 MT-QC plasmids, respectively. (PDF) [file pone.0145698.s002.pdf]

Figure S2. Amplification curves of real-time PCR with and without WTB targeting each of the 12 missense mutations at *KRAS* codon 12 and 13

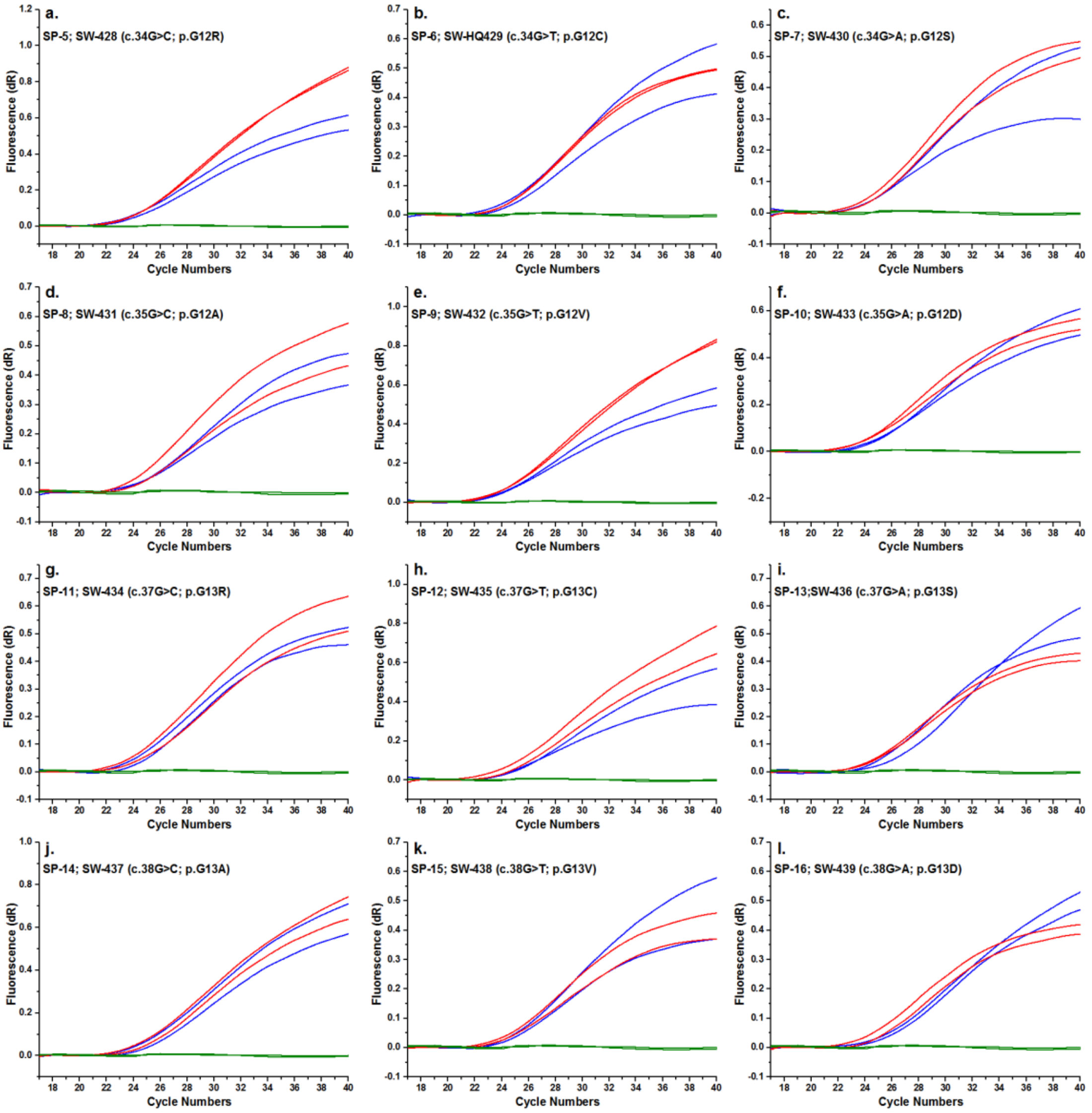

Pane a to l show the amplification curves of real-time PCR with and without WTB targeting specific MT-alleles at *KRAS* codons 12 and 13 as indicated (SP-5 to -16 QC plasmid). The target-specific primers and corresponding mutation information are indicated in each panel of the image. The blue and red line curves indicate PCR with and without WTB, respectively. The olive lines indicate the results from the NTC mixture.
