# Supplementary material for: Single-Tubed Wild-Type Blocking Quantitative PCR Detection Assay for the Sensitive Detection of Codon 12 and 13 KRAS Mutations
Source: PLoS One. 2015 Dec 23;10(12):e0145698. doi: 10.1371/journal.pone.0145698 (PMC4689371; doi:10.1371/journal.pone.0145698)
Supplement: S3 Fig — Panel a to i show the amplification curves of samples containing specific c.35G>A allele mutation percentages of 100%, 50%, 25%, 10%. 1%, 0.1%, 0.02%, 0.01%, 0%, respectively. (PDF) [file pone.0145698.s003.pdf]

Figure S3. Sensitivities of real-time WTB-PCR

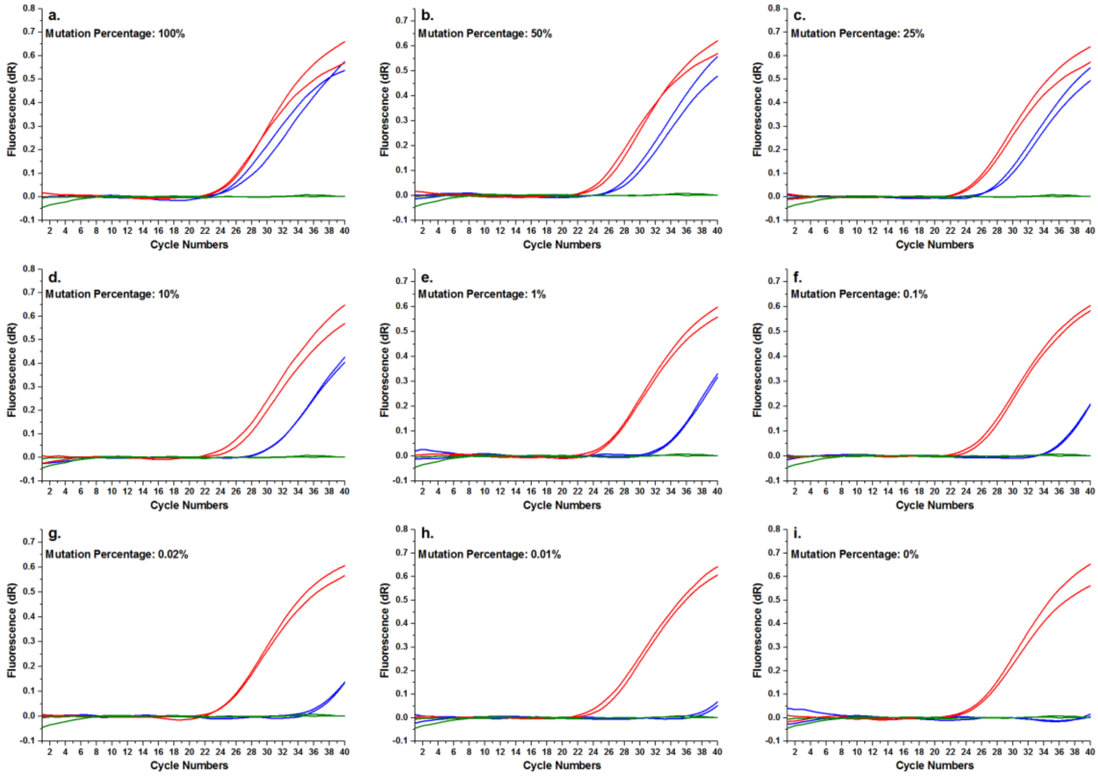

Pane a to i show the amplification curves of real-time PCR with and without WTB, containing specific c.35G>A allele mutation percentages. The blue and red line curves indicate PCR with and without WTB, respectively. The olive lines indicate the results from the NTC mixture.
