# Supplementary material for: Single-Tubed Wild-Type Blocking Quantitative PCR Detection Assay for the Sensitive Detection of Codon 12 and 13 KRAS Mutations
Source: PLoS One. 2015 Dec 23;10(12):e0145698. doi: 10.1371/journal.pone.0145698 (PMC4689371; doi:10.1371/journal.pone.0145698)
Supplement: S1 Table — All targeted sequences overlapping with WTB are listed. And only mutated bases that were observed following sequencing results are shown. (PDF) [file pone.0145698.s004.pdf]

Table S1. Sequencing results of amplicons generated after 55 amplification thermal cycles#

Sample	Without	With WTB												
ID	WTB	30A	31G	32C	33T	34G	35G	36T	37G	38G	39C	40G	41T	42A
T10	c.35G/A						A							
T11	WT					G/A	G/A		G/A					
T12	c.34G/C					C								
T13	WT					G/A	G/A		G/A					
T24	WT					G/A	A/G							
T40	C.38G/A									A				
T44	WT					G/A	A/G		G/A	G/A				
T49	WT					G/A	C/G/A		G/A					
T53	c.35G/A						A							
T58	WT					G/A	A/G							
T59	WT						G/A		G/A					
T60	WT					G/A	G/A		C/G/A/			G/A		
T64	WT					A/G	G/A							
T66	c.35G/T						T							
T67	c.35G/A						A							
T69	WT					G/A	C/G/A							
T71	WT					G/A	T/G							
T72	WT					G/A	G/A		G/A					
T73	WT					G/A	A/G		G/A					
T101	WT					G/A	G/A		G/A					
T102	c.38G/A									A				
T103	WT					G/A	G/A		G/A					
T104	WT					G/A	G/A		G/A					
T105	WT					G/A	G/A		A/G					
T106	c.34G/T					T								
T107	WT					G/A	C/G/A		G/A					
T108	WT					A/G			G/A					
T109	WT					G/A	G/A		G/A					
T110	WT					G/A	G/A		G/A					
T111	WT					G/A			G/A					
T112	c.35G/A						A							
T113	WT					A/G	G/A		G/A					
T114	c.38G/A									A				
T115	c.38G/A									A				
T116	c.38G/A									A				
T117	WT					G/A	G/A		G/A					
T118	WT					G/A				G/A				
T119	WT						G/A		G/A					

**T120**	**WT**								**T/G/A/**	**G/A**				
**T121**	**c.35G/C**						**C**							
**T122**	**c.38G/A**									**A**				
**T123**	**c.38G/A**									**A**				
**T124**	**c.35G/C**						**C**							
**T125**	**c.35G/A**						**A**							
**T126**	**c.35G/A**						**A**							
**T127**	**WT**					**G/A**	**T/G/A/**							
**T128**	**c.37G/T**								**T**					
**T129**	**WT**					**G/A**	**G/A**			**G/A**				
**T130**	**WT**					**G/A**	**G/A**		**G/A/C**	**G/A**	**A**			

#In the preliminary experiments, all samples were amplified using real-time PCR in reaction mixtures with or without 0.5 μM WTB, and 55 thermal amplification cycles. In the “with WTB” column, all targeted sequences overlapping with WTB are listed. Underlined letters indicate the bases complementary to residual LNA in WTB oligonucleotides. Only mutated bases that were observed following sequencing results are shown. The order of bases indicates the order of associated with peak heights. For example, A/G indicates that the peak of A base was higher than that of the G base. All confirmed mutated bases in reactions that were subjected to only 40 cycles are highlighted in yellow.
